# Laparoscopic management of an uncommon cause for right lower quadrant pain: A case report

**DOI:** 10.1186/1757-1626-1-164

**Published:** 2008-09-19

**Authors:** Alfie J Kavalakat, Chalissery J Varghese

**Affiliations:** 1 Consultant surgeon, Center for Laparoscopic Surgery and Minimally invasive procedures, Westfort Hospital & Westfort Hi-tech Hospital, Thrissur 680004, Kerala, India

## Abstract

**Introduction:**

Primary segmental infarction of the greater omentum is an infrequent cause for right lower quadrant pain. The exact aetiology is unknown and the right side is more commonly involved. It usually presents like acute appendicitis and the diagnosis is made during exploration.

**Case report:**

We report such a case which was diagnosed and managed by laparoscopy. A 27-year-old male presented with features suggestive of acute appendicitis. Preoperative imaging failed to diagnose the condition. Laparoscopy showed a segment of oedematous and haemorrhagic greater omentum adherent to the parietal wall over the right lower quadrant. The infarcted segment was excised and removed in a non permeable bag through the umbilical port. A short edited video of the operative findings and the procedure executed is also provided.

**Conclusion:**

Primary segmental infarction of the greater omentum is an uncommon cause of right lower quadrant pain mimicking appendicitis. Laparoscopy is both diagnostic as well as therapeutic.

## Introduction

Idiopathic segmental infarction of the greater omentum is a rare cause of acute abdominal pain, which was first described by Bush in 1896[1]. It is a disease of unknown aetiology and the right side of omentum is involved in 90% of the cases, mimicking acute appendicitis in the majority [2]. Even though sonographic and CT findings of the condition has been described, diagnosis is usually made during surgical exploration. The value of laparoscopy in the diagnosis and therapy of non-specific acute abdominal pain is well known [3]. Laparoscopy enables the surgeon to inspect the entire abdominal cavity, which is an advantage over a limited open access (McBurney incision) in suspected appendicitis. Laparoscopy is both diagnostic as well as therapeutic for this condition.

## Case presentation

A 27-year-old male, non-resident Indian working in Qatar presented with right lower quadrant pain of one week duration. The pain was acute in onset, not very severe and was aggravated by movements. He was not having fever, nausea or vomiting. He had no urinary symptoms and the bowel movements were normal. He was on oral medications prescribed by the local physician and was having loss of appetite. On presentation his pulse rate was 86/mnt, BP was 130/80 mm of Hg. and temperature 98.6°F. Abdomen was soft and he had tenderness in the right lower quadrant, with rebound tenderness. Blood tests showed a total white blood cell count of 11200/mm^3 ^(with 82% Neutrophils and 28% Lymphocytes) and the routine biochemical investigations were within normal limits. Ultra sonogram of the abdomen failed to visualize the appendix but the patient had probe tenderness in the right iliac fossa.

**Figure 1:**
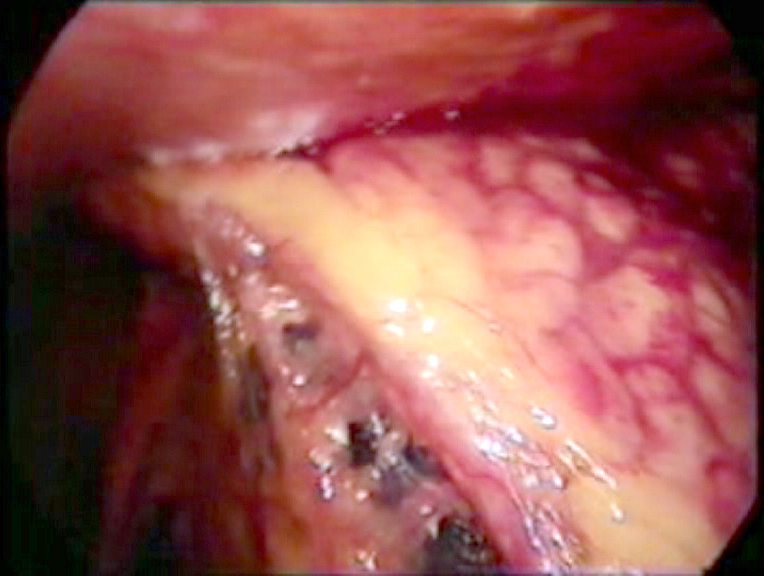
The involved segment of greater omentum adherent to the parietal wall on the right side.

**Figure 2:**
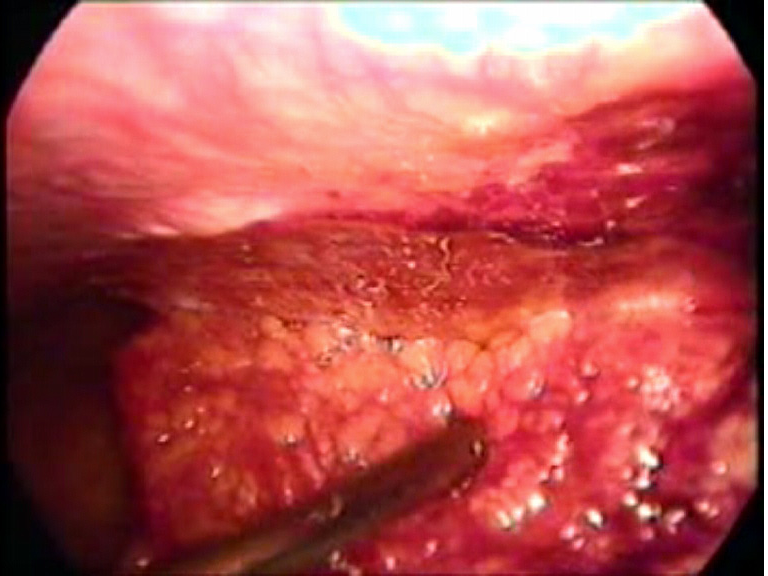
The infarcted omental segment being detached from the parietal wall using a suction-irrigation cannula.

**Figure 3:**
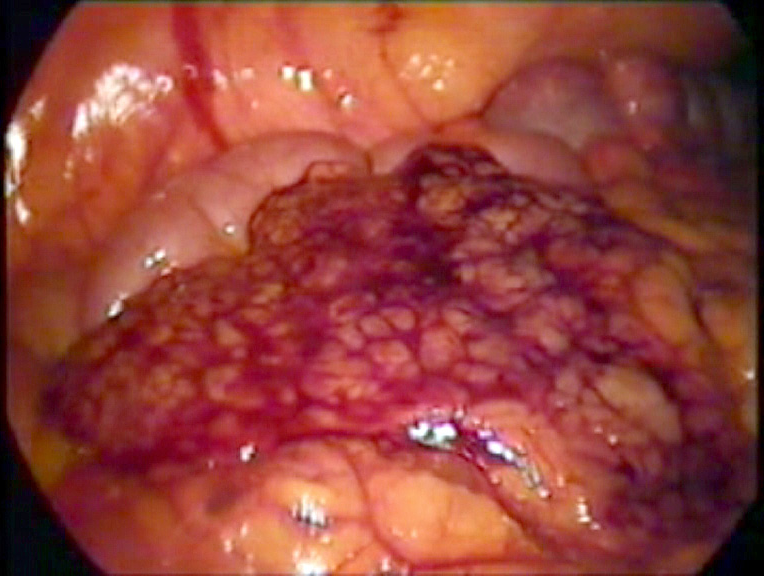
The detached segment of infracted greater omentum lying over the bowel.

With a clinical diagnosis of appendicitis laparoscopy was done. One 10 mm port for camera at the umbilicus and two 5 mm working ports one in each iliac fossa were used. It showed a segment of ischemic omentum stuck to the parietal wall in the right lower quadrant (figure 1). Appendix was normal. The segment of omentum was detached from the parietal wall (figure 2 &3) and excised. The excised segment was delivered in an endobag through the umbilical port (See additional file 1: Omental Infarction Video for an edited version of the laparoscopic findings and the procedure done). The post operative period was uneventful and he was discharged on the second post operative day. Histopathological evaluation showed focal ischemic necrosis and granulation tissue.

## Discussion

Primary segmental infarction of the greater omentum is rare, with a reported incidence of 0.1% of the total laparotomies performed for acute abdomen [4]. The exact aetiology is unknown. The clinical picture simulates appendicitis, diverticulitis, or cholecystitis depending on the site of infarction [5]. The right side is more commonly involved and the most common preoperative diagnosis is acute appendicitis.

Conservative management has been proposed when a correct diagnosis is made preoperatively and the patient is stable [6]. But the diagnosis is usually made at laparoscopy or laparotomy. Through a limited right iliac fossa incision the diagnosis is difficult and even if diagnosed it would necessitate making a larger or complementary incision for excision of the infracted omentum. Laparoscopic approach is safe and effective in managing idiopathic segmental infarction of greater omentum and is associated with low morbidity [7-9]. With the popularity of minimal access surgery increasing, more cases are being documented and reported. We would like to emphasize the usefulness of laparoscopy in patients with right lower quadrant pain, which is both diagnostic as well as therapeutic.

## Consent

Written informed consent was obtained from the patient for publication of this case report and accompanying images. A copy of the written consent is available for review by the Editor-in-Chief of this journal.

## Competing interests

The authors declare that they have no competing interests.

## Authors' contributions

AJK was involved in collection of data and drafting the manuscript. CJV was involved in preparing the manuscript and critical evaluation. Both authors read and approved the final manuscript.

## Supplementary Material

Additional file 1 Omental Infarction video. An edited version of the original video of operative findings and the operative procedure. Click here for file
